# Adherence to 24-hour movement guidelines and associations with mental well-being: a population-based study with adolescents in Canada

**DOI:** 10.1186/s12889-025-21857-7

**Published:** 2025-03-06

**Authors:** Eva Oberle, Suiqiong Fan, Tonje M. Molyneux, Xuejun Ryan Ji, Mariana Brussoni

**Affiliations:** 1 Human Early Learning Partnership, School of Population and Public Health, Faculty of Medicine, The University of British Columbia, 2206 East Mall, Vancouver, BC V6T 1Z3 Canada; 2 Department of Educational and Counselling Psychology, and Special Education, Faculty of Education, The University of British Columbia, Vancouver, Canada; 3 Department of Pediatrics, Faculty of Medicine, The University of British Columbia, Vancouver, Canada

**Keywords:** 24-hour movement guidelines, Mental well-being, Adolescence, Sex differences, Screen time, Physical activities, Sleep, Population-level research

## Abstract

**Background:**

Insufficient physical activity, excessive recreational screen time, and inadequate sleep pose health risks in school-aged children and adolescents. The Canadian 24-Hour Movement Guidelines for Children and Youth advocate for balanced daily movement behaviours. This population-level study in British Columbia (BC), Canada, examined the proportion of young adolescents following these guidelines and how adherence correlated with their mental well-being.

**Methods:**

Using cross-sectional self-report data from 26,974 grades 6–8 children (48% girls, mean age = 13.31) who had completed the Middle Years Development Instrument (MDI) in BC in 2023, we calculated the percentages of children meeting physical activity (at least 1 h daily), sleep (9 + hours for 5- to 13-year-olds, 8 + for > 13-year-olds), and screen time (no more than 2 h daily) recommendations. Eight groupings were created, indicating how many and which movement behaviour guidelines were met: none, sleep only, physical activity only, screen time only, physical activity and sleep, physical activity and screen time, sleep and screen time, and all three behaviours. Mean differences in depressive symptoms, optimism, and satisfaction with life scores across categories were estimated through sex-stratified linear mixed models, adjusting for covariates.

**Results:**

Four percent of adolescents met all recommendations, while 15% met none. Meeting some or all recommendations was associated with higher levels of optimism and satisfaction with life, and lower levels of depressive symptoms compared to meeting none. Beneficial effects were overall larger for girls, and largest for depressive symptoms among girls meeting all recommendations versus none (-1.05, 95% CI [-1.14, -0.96]).

**Conclusions:**

Adherence to the Canadian 24-Hour Movement Guidelines was associated with higher levels of well-being, but most grades 6–8 adolescents in our study did not meet the recommendations. Given sex differences in meeting the movement behaviour recommendations, public health strategies need to consider targeted interventions aimed at improving adherence to these guidelines, particularly focusing on reducing recreational screen time and increasing physical activity.

**Supplementary Information:**

The online version contains supplementary material available at 10.1186/s12889-025-21857-7.

The health benefits of movement are well-established for children and adolescents [1]. However, modern lifestyles have become increasingly sedentary, which has been linked to heightened health risks [2], especially for screen-based sedentary behaviour [3]. To address this public health concern, Canada developed the *24-Hour Movement Behaviour Guidelines for Children and Youth* (referred to as the 24-Hour Guidelines hereafter) [4]. The guidelines offer a framework for adolescent health promotion and include recommendations for physical activity (PA; light, and moderate to vigorous), sedentary behaviour (including recreational screen time), and sleep across a 24-hour period [5]. Some studies have examined the impact of children and adolescents meeting these movement behaviour guidelines on health [6, 7]. However, research has primarily focused on the impact of one behaviour rather than the additive or combined effects of several. Moreover, the extant research has focused on physical health outcomes (e.g., obesity), and fewer studies have examined mental well-being and used population-level samples [7, 8]. Especially positive well-being, an important focus of strength-based adolescent development approaches, has received less attention [9]. This study addresses these gaps by exploring how combinations of adherence to movement behaviour guidelines were associated with positive and negative indicators of adolescent mental well-being, considering differences by sex.

## Movement guidelines for children and adolescents

Canada’s 24-Hour Guidelines integrate recommendations for sleep, sedentary behaviour, and physical activity—collectively referred to as movement behaviours—based on their impact on health [5]. For school-aged children and adolescents, ≥ 60 min per day of moderate-to-vigorous physical activity for 5- to 17-year-olds are recommended; ≤ 2 h per day of recreational screen time; and 9 to 11 h per day of sleep for 5- to 13-year-olds and 8 to 10 h per day of sleep for 14- to 17-year-olds. Similar recommendations exist in other countries (e.g., South Africa, Australia) as well as from the World Health Organization [10]. 

Several studies have examined adherence to these guidelines in Canada. For example, Janssen and colleagues [7] reported only 3% of a representative sample (*N* = 22,115) of Canadian children and adolescents (10–17 years old) met all three movement behaviour recommendations; 25% met two, 51% met one, and 21% met none. Adherence was highest for sleep (66%) followed by physical activity (35%) and screen time (8%). Roberts et al. [11] found similar results; 17.5% of their representative sample (*N* = 3,111) met all three recommendations and the younger age group (5–11 years old) and boys were more likely to meet the recommendations compared to older children (12-17 years old) and girls. Furthermore, Carson et al. [6] found that 17% of their representative sample (*N* = 4,157) of Canadian children and adolescents (6–17 years old) met all three recommendations, and this group demonstrated better health indicators (body mass index, waist circumference, blood pressure, behaviour, and aerobic fitness) than those who did not meet the recommendations. A dose-response pattern was evident, with health benefits being larger when more recommendations were met.

## Relations between movement behaviours and adolescent well-being

Evidence linking the movement behaviours in the 24-Hour Guidelines (PA, screen time, and sleep) with mental well-being is growing. Several systematic reviews have documented the associations between adolescent PA with better mental health outcomes [12, 13]. For example, Biddle et al. [12]. found that adolescent PA was linked to reductions in depression and anxiety, with emerging evidence suggesting psychosocial, behavioural and neurobiological mechanisms that partially explained the link between PA and depression. Similarly, a systematic review of 114 studies by Rodriguez-Ayllon and colleagues (2019) found a significant effect of adolescent PA on mental health (e.g., lower levels of depression and higher levels of satisfaction with life) [13]. A recent study suggests long-term benefits of PA on mental health [14]; specifically, higher levels of physical activity at ages 12–13 were associated with lower levels of emotional problems at ages 15–16 in a cohort of young adolescents in the UK. Despite these recent advances in research, more large-scale and higher-quality studies are needed to confirm the mental health benefits of PA for adolescents [12]. 

Sedentary behaviour includes any low-energy activity performed while awake, often seated or in a reclined position [15]. For many adolescents, sedentary behaviour occurs while engaging in screen-based activities. A review of adolescent sedentary behaviour surveillance data in Canada from 2007 to 2017 found that while time spent on television and video viewing remained stable over the decade, time spent using recreational computers or electronic devices increased by one to two hours daily [16]. Some studies found sex differences in excessive screen time (i.e., > 2 h per day) but findings are inconsistent as to whether this is more common in boys or girls [17, 18]. High levels of screen time are a concern as they have been associated with negative health and well-being outcomes among adolescents globally [19]. A systematic review of over 200 studies found that lower levels of screen time were associated with positive health indicators whereas excessive screen time was associated with negative outcomes including lower fitness, self-esteem, and academic achievement [2]. A recent systematic review of associations between sedentary behaviour and adolescent (ages 10–19 years) mental health also found strong evidence linking depressive symptoms to excessive screen time (i.e., > 2–3 h per day) [20, 21]. The authors reported sex differences, with girls more likely than boys to report depressive symptoms at higher screen time levels [22, 23]. Further, research exploring relations between leisure time, physical activity, sedentary behaviour, and symptoms of depression and anxiety in a population-based sample (*N* = 9,702) of adolescents in Canada found that both physical inactivity (defined as being physically active less than once a month) and sedentary behaviour (defined as 2+ hours/dayof sedentary activity) were significantly related to symptoms of depression and anxiety when modeled separately [24]. However, in joint models, sedentary behaviour did not emerge as consistently related to these symptoms suggesting that these two behaviours need to be distinguished in future research. In British Columbia (BC), Canada, Oberle and colleagues found that screen time > 2 h per day was linked to lower levels of life satisfaction and optimism, and higher levels of anxiety and depressive symptoms in grade 7 students, with stronger effects for girls compared to boys [25]. 

Sufficient sleep is important for young people’s health [26], especially to support healthy brain development [27]. Achieving the recommended number of hours of sleep has become more challenging for today’s youth potentially due to technology and screen use, stress, and artificial lights [28]. Recent cross-sectional and longitudinal studies suggest a relationship between sleep and health indicators. Chaput and colleagues analyzed 141 studies on the relationship between sleep duration and health indicators for children and adolescents finding collective evidence that shorter sleep duration was associated with poorer physical and mental health [29]. 

More recent research has explored combinations of sleep duration, physical activity, and sedentary time in association with mental health problems for children and adolescents. Sampasa-Kanyinga et al. analyzed 13 cross-sectional studies and found that when all three recommendations were met, children and adolescents had better indicators of mental health compared to those meeting none [30]. A dose-response relationship was evident suggesting that with an increasing number of recommendations met, mental health indicators improved [30]. However, the quality of evidence was low in several studies, indicating a need for more research to explore these associations.

## The present study

This study examined the proportion of young adolescents adhering to 24-Hour Guidelines guidelines in BC, and associations between adherence to one or multiple 24-Hour Guidelines and positive and negative mental well-being outcomes. Drawing from a population-based sample of grade 6–8 students, we examined two main research questions: (1) What percentage of adolescents adhered to each of the 24-Hour Guidelines and to different combinations of them, and does this differ for boys and girls?, and (2) To what extent was adherence to one or multiple of the recommendations associated with higher levels of satisfaction with life and optimism, and lower levels of depressive symptoms in boys and girls? Based on previous research [2, 30], we hypothesized that: (1) meeting none of the recommendations would be associated with lowest levels of mental well-being (2), meeting all of the recommendations would be associated with highest levels of mental well-being, and (3) meeting one or two recommendations would be positively associated with well-being, increasing stepwise with more recommendations met. Examining the percentage of boys and girls meeting each of the recommendations and combinations thereof, and whether there were sex differences in the associations were exploratory questions.

## Methods

### Participants

This study was based on cross-sectional data from 26,974 students (48% female) in grades 6–8 (range: 11–16 years; mean age = 13.31 years, standard deviation (SD) = 0.88), who completed the Middle Years Development Instrument (MDI) survey during the 2022/2023 school year. The MDI is a child and adolescent self-report survey that is implemented annually at a population-level in participating school districts in BC (N_districts_ = 37, N_schools_ = 431 in 2022/2023). The student participation rate in school districts varied from 59% to 89% (Mean = 81%, SD = 0.09). The distribution of first language learned by participants in the study reflected the diversity of the population in the province: 77% of participants reported English as their first language learned, 7% reported Mandarin, 6% reported Punjabi, and 5% reported Cantonese. Other first languages learned (< 5%) were Spanish, Tagalog, and French. Schools in rural/small (population < 29,999), medium (population 30,000–99,999), and large (population 100,000 or greater) population centres in BC [31] were represented in the study. Detailed methods for the MDI have been documented elsewhere (Schonert-Reichl et al., 2013; Thomson et al., 2018). See Table 1 for sample characteristics.

**Table 1:** Descriptive statistics for the total and sex-specific samples

Sample characteristics^a^		Overall	Girls	Boys
N		26,974	13,053 (48%)	13,921 (52%)
Age, mean (SD)		13.31 (0.88)	13.28 (0.88)	13.33 (0.87)
Optimism, mean (SD)		3.46 (0.95)	3.31 (0.97)	3.59 (0.92)
	Missing^b^	790 (3%)	365 (3%)	425 (3%)
Satisfaction with life, mean (SD)		3.64 (0.97)	3.46 (1.01)	3.80 (0.90)
	Missing^b^	1508 (6%)	771 (6%)	737 (5%)
Depressive Symptoms, mean (SD)		3.02 (1.01)	3.25 (1.00)	2.81 (0.97)
	Missing^b^	825 (3%)	427 (3%)	398 (3%)
SES index 2016, mean (SD)		110.63 (13.22)	110.73 (13.18)	110.53 (13.25)
School type	Elementary	8802 (33%)	4294 (33%)	4508 (32%)
	Middle	9368 (35%)	4530 (35%)	4836 (35%)
	High school	6009 (22%)	2820 (22%)	3187 (23%)
	K12 combined	2805 (10%)	1409 (11%)	1390 (10%)
First language is English	No	5759 (21%)	2787 (21%)	2972 (21%)
	Yes	20,874 (77%)	10,117 (78%)	10,757 (77%)
	Missing^b^	341 (1%)	149 (1%)	192 (1%)
Grade	Grade 6	7114 (26%)	3538 (27%)	3576 (26%)
	Grade 7	5160 (19%)	2493 (19%)	2667 (19%)
	Grade 8	14,700 (55%)	7022 (54%)	7678 (55%)
Daily physical activity	Not met	18,715 (69%)	9887 (76%)	8828 (63%)
	Met	7046 (26%)	2656 (20%)	4390 (32%)
	Missing^b^	1213 (5%)	510 (4%)	703 (5%)
Sleep ( > = 9 h for 5–13 years, >=8 for 14–17 years)	Not met	6299 (23%)	3524 (27%)	2775 (20%)
	Met	19,755 (73%)	9192 (70%)	10,563 (76%)
	Missing	920 (3%)	337 (3%)	583 (4%)
Screen time < = 2 h	Not met	21,824 (81%)	10,263 (79%)	11,561 (83%)
	Met	3814 (14%)	2248 (17%)	1566 (11%)
	Missing^b^	1336 (5%)	542 (4%)	794 (6%)
Meeting combinations of 24-hour movement recommendations	None met	4086 (15%)	2437 (19%)	1649 (12%)
	Activity only	1429 (5%)	622 (5%)	807 (6%)
	Sleep only	11,517 (43%)	5555 (43%)	5962 (43%)
	Screen only	269 (1%)	186 (1%)	83 (1%)
	Activity and sleep	4057 (15%)	1313 (10%)	2744 (20%)
	Activity and screen	176 (1%)	100 (1%)	76 (1%)
	Sleep and screen	2142 (8%)	1373 (11%)	769 (6%)
	All met	1094 (4%)	518 (4%)	576 (4%)
	Missing	2204 (8%)	949 (7%)	1255 (9%)
Number of recommendations met	None	4086 (15%)	2437 (19%)	1649 (12%)
	One	13,215 (49%)	6363 (49%)	6852 (49%)
	Two	6375 (24%)	2786 (21%)	3589 (26%)
	Three	1094 (4%)	518 (4%)	576 (4%)
	Missing^b^	2204 (8%)	949 (7%)	1255 (9%)

*Notes*

^a^Variable distributions are reported as n (%) unless otherwise stated. Percentages may not add up to 100 due to rounding

^b^Missing categories are reported as n (%)

### Procedure

Data were collected between January and March 2023. The MDI was administered electronically by school personnel during the school day, following a detailed implementation manual and training materials provided beforehand. Students had the opportunity to ask questions during the survey. An informed passive consent procedure was employed. Families were informed about the study. All students participated unless their guardians withdrew them, they did not provide assent, or they were absent during the survey implementation. All participants provided assent before completing the survey. This study was approved by the University of British Columbia behavioural ethics review board (#H18-00507) and the administrations of the involved school districts. Funding was provided by the Social Sciences and Humanities Research Council of Canada.

### Measures

All self-report measures were derived from the MDI, a self-report questionnaire designed to examine well-being, health, extracurricular activities, and school-related experiences among children and adolescents. For detailed information on the MDI, including its creation, adjustments to scales, and validation, refer to previous work [32]. In this study, we focused on measures capturing the movement behaviours specified in the Canadian 24-Hour Movement Guidelines for Children and Youth [4, 5], mental well-being indicators and demographic variables.

#### Demographic information

Participants self-reported their first language learned (English = 20,874, other language = 5,759). Age and sex data were obtained from school records. Age was available rounded to the nearest one. Information about sex (male = 13,921, female = 13,053) was based on official records (e.g., birth certificates) presented at the time of school registration. Ten participants with missing sex information were excluded. Females will be referred to as girls and males will be referred to as boys for the remainder of this study.

We utilized a BC-specific version of the Canadian Neighbourhoods Early Child Development Socioeconomic Status Index (neighbourhood SES index) [33]. Individual-level SES was not available. Developed in 2006 and updated with each census, the index comprises seven variables reflecting social determinants of health, such as poverty, wealth, income, education, and family structure [33]. The SES index in the current study was calculated based on the 2016 census data. In past research, the SES index has explained a significant portion of variance in neighbourhood-level vulnerability in child developmental health, outperforming other commonly used Canadian indices [34]. 

#### Positive and negative mental well-being indicators

Adolescents’ self-reported satisfaction with life (SWL) was measured with a 5-item scale (Cronbach’s alpha of 0.87; example item: “In most ways, my life is close to the way I want it to be”) and optimism was measured with a 3-item scale (Cronbach’s alpha of 0.78; example item: “I start most days thinking that I will have a good day”) [35, 36]. Depressive symptoms were measured with a 3-item scale (Cronbach’s alpha of 0.78; example item: “I feel unhappy a lot of the time”) [37]. Responses for all mental well-being indicators were rated on a 5-point Likert scale (1 = strongly disagree, 2 = disagree, 3 = neither agree nor disagree, 4 = agree, 5 = strongly agree). Average item scores for each scale were considered as outcome variables in the analyses.

#### Measuring adherence to 24-Hour guidelines

**Physical Activity.** Physical activity was assessed with a one-item measure asking adolescents how many days in a normal week they are physically active for at least 60 min in a way that increases their heart rate and makes them out of breath some of the time. A brief description of when physical activity may take place was provided (e.g., sports, school activities, playing with friends, walking to school) and a list of examples was given (e.g., running, biking, fast walking, dancing, soccer, skating). Response options ranged from *Never*, *1 day*, *2 days*,…, to *every day*. Aligning with the 24-Hour Guidelines, responses indicating “every day” were considered having met the recommendation (1 = met, 0 = not met). Previous research examined the re-test reliability and concurrent validity of a similarly-worded single-item PA measure for adolescents by comparing it with a multi-item questionnaire and accelerometer output [38]. Findings supported the reliability and validity of the single-item PA measure.

**Recreational Screen Time.** Adolescents reported their typical daily recreational screen time for three screen-based activities: (1) playing video or computer games, (2) sitting and watching TV, movies, or videos, including YouTube, and (3) spending time on social media, sites or apps, such as Instagram, Snapchat, Twitter, Facebook, TikTok either browsing or posting. For each screen time activity per day, response options were: (1) I do not do this activity; (2) less than 1 h; (3) 2–3 h; (4) 3–4 h; (5) 4–5 h; (6) 5 h or more. When screen time was under 2 h for an activity, we took the interval midpoint for the activity (i.e., never = 0; less than 1 h = 0.5, 1–2 h = 1.5). We then created a summative indicator for total screen time across all recreational screen time activities. Aligning with the 24-Hour Guidelines, up to 2 h per day of total screen time was considered as having met this recommendation (1 = met, 0 = not met).

**Sleep Duration.** Adolescents reported their typical bedtime (before 9 pm, 9–10 pm 10–11 pm, 11 pm– 12 am, or after 12 am) and wake-up time (before 6 am, 6–7 am, 7–8 am, or after 8 am) during weekdays. Sleep duration was computed by determining the midpoint of each time range (e.g., 9–10 pm = 9:30 pm) and then subtracting them. Consistent with the 24-Hour Guidelines [4], for adolescents up to the age of 13 at the time of MDI implementation (*N* = 12,213), we considered a minimum of 9 h of sleep as meeting the recommendation (1 = met, 0 = not met). Consistent with the age-specific recommendations in the 24-Hour Guidelines, for adolescents aged 14 and older (*N* = 14,761), we used 8 h as the cut-off. We did not consider excessive sleep in this study, as the effect of too much sleep on health is less consistent, and the mechanism might be different from inadequate sleep [29]. 

To examine the relationships between movement behaviours and mental well-being outcomes, we grouped adolescents into eight non-overlapping categories: (1) adolescents who met none of the recommendations; (2) adolescents who met the physical activity recommendation only; (3) adolescents who met the screen time recommendation only; (4) adolescents who met the sleep recommendation only; (5) adolescents who met the physical activity and screen time recommendations; (6) adolescents who met the physical activity and sleep recommendations; (7) adolescents who met the screen time and sleep recommendations; and (8) adolescents who met all recommendations.

### Analytic approach

First, descriptive statistics were calculated for the demographic variables, mental well-being outcomes and movement behaviours based on the 24-Hour Guidelines. Missingness was examined for all variables. For RQ1, we first computed the percentage of adolescents meeting the recommendations for physical activity, recreational screen time use, and sleep duration separately and for all possible combinations. We also computed the percentage of adolescents meeting overall no, one, two, or all recommendations (see Table 1). Findings are reported for the overall sample and by sex.

For RQ2, we first visually displayed mental well-being outcomes in relation to different movement behaviour combinations, before adjusting for other covariates. We then estimated the difference in each mental well-being outcome between adolescents who adhered to some or all of the movement behaviour recommendations and those who did not meet any of the recommendations, using linear mixed models. To examine the variability in well-being outcomes due to the school in which participants were nested, intraclass correlation coefficients (ICCs) were computed. A nested approach reflects the data structure in the present study; it prevents underestimating standard errors and an increased risk of Type 1 error. Schools were treated as clusters and random intercepts were estimated. We stratified the analyses by sex, and all models were adjusted for age, grade, and neighbourhood SES. There were 10% of participants with missing data in the exposure and/or outcome variables. Demographic variables were similar across participants with and without missing data, except for grade (see supplementary material). Mixed models have been shown to be effective in handling missing data in the clustered data setting without conducting multiple imputations [39]. When missing at random can be assumed, mixed models use full information maximum likelihood methods to obtain unbiased estimates [39, 40]. To test the robustness of mixed methods in our analyses, we conducted multiple imputation using multiple imputation by chain equations (m = 10) and compared the findings with our main results for depressive symptoms (see supplementary material) in our preliminary analyses [39, 41, 42]. The results overall were consistent, supporting the use of mixed methods in our analyses. Finally, for the exploratory analysis in sex differences, we plotted sex-specific adjusted mean differences to illustrate the sex difference in the effect of different movement behaviour combinations. Analyses were conducted using nlme and ggplot2 packages in R [43–45]. 

## Results

### Adherence to single and multiple 24-Hour guidelines (RQ1)

Table 1 shows the percentages of adolescents meeting each of the 24-Hour Guidelines. In total, 73% of adolescents met the sleep duration recommendation (76% of boys, 70% of girls), 69% met the daily physical activity recommendation (76% of boys, 63% of girls), and 14% of adolescents met the screen time recommendation (17% of girls, 11% of boys). Overall, 4% of the boys and girls met all three movement behaviour recommendations and 15% of adolescents met none (19% of girls, 12% of boys) (Table 1). For single-met recommendations, meeting only the sleep recommendation was most common (43% of boys and girls) and meeting only the screen time recommendation was least common (1.4% of girls, 0.6% of boys). Percentages for combinations of recommendations met tended to be low when they included screen time (meeting physical activity + screen time recommendations: 0.8 of girls, 0.5% of boys; meeting sleep + screen time recommendations: 11% of girls, 6% of boys).

### Associations between movement behaviours and mental well-being (RQ2)

**Fig. 1:**
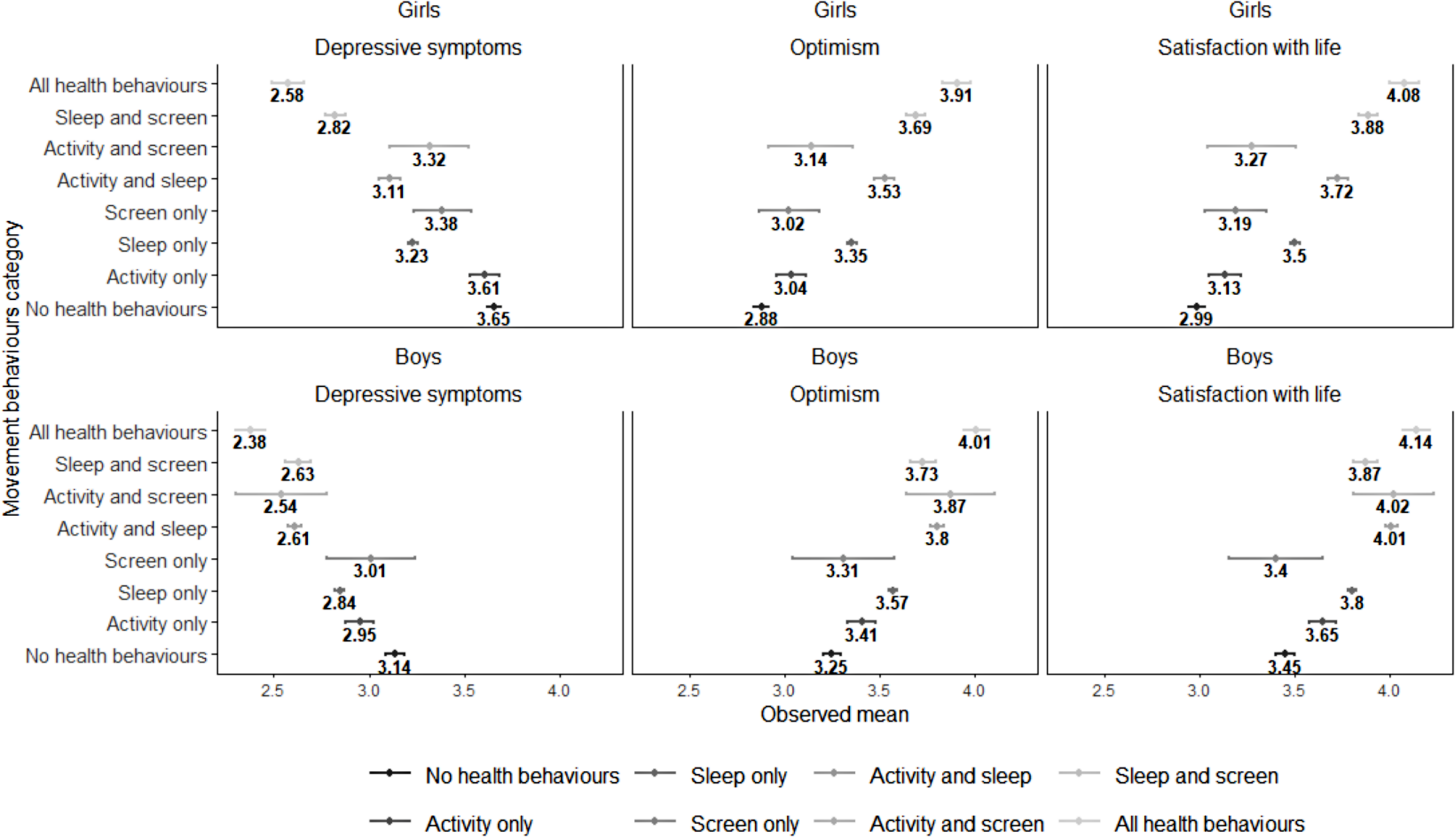
Sex-specific observed means and CIs for each outcome. Notes. Unadjusted means and 95% CIs are presented. Upper panel includes mean scores for depressive symptoms, optimism, and SWL for girls. Lower panel includes mean scores for depressive symptoms, optimism, and SWL for boys

**Fig. 2:**
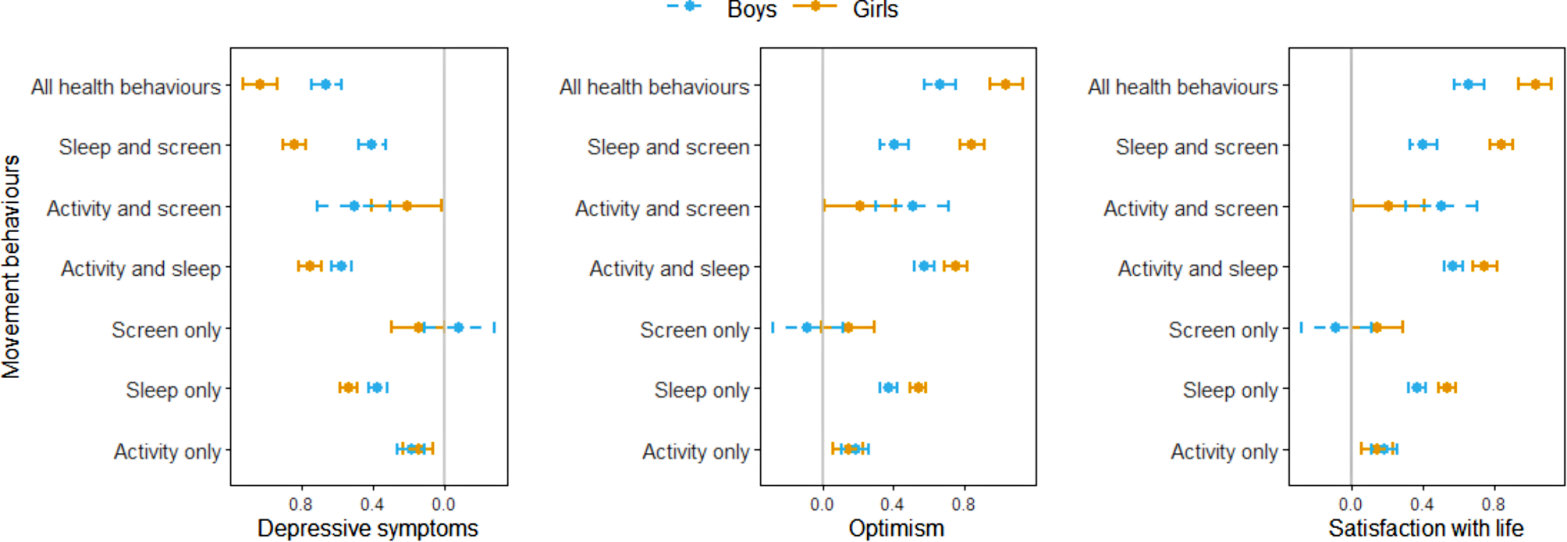
Sex-specific adjusted mean differences in mental well-being, comparing the well-being of adolescents in different movement behaviour categories to the well-being of adolescents who did not meet any of the movement behaviour recommendations. *Notes.* Six regression analyses were conducted (for boys and girls) for depressive symptoms, optimism, and SWL scores. The vertical grey line represents a null effect. Bars further away from the line indicate a larger difference in well-being outcomes in a positive way, compared to adolescents who did not meet any of the movement behaviours

Observed means and 95% confidence intervals (CIs) of each outcome by sex are displayed in Fig. 1 for different movement behaviour categories. Results from linear mixed models are shown in Table 2. ICCs ranged from 0.01 to 0.02, indicating low variability between schools. Using ‘meeting none of the movement behaviour recommendations’ as the reference group, sex-specific adjusted mean differences for each movement behaviour category are illustrated in Fig. 2a-c. After adjusting for age, grade, and neighbourhood SES, meeting one or more recommendations in the 24-Hour Guidelines was associated with lower depressive symptoms, higher SWL scores, and higher optimism scores, among both boys and girls. The largest association was observed among girls with all behaviours versus those with none, with the largest effect for depressive symptoms (-1.05, 95% CI [-1.14, -0.96]), followed by SWL (1.03, 95% CI [0.94, 1.13]), and optimism (0.99, 95% CI [0.90, 1.07]). For categories reflecting that adolescents met two of three recommendations, we found similar positive relationships between these categories and well-being outcomes. We did not observe a significant association between meeting only the screen time recommendation and mental well-being.

**Table 2:** Associations between mental well-being outcomes and movement behaviours from sex-specific linear mixed models

Est.	Depressive Symptoms		Optimism		Satisfaction With Life	
	Girls	Boys	Girls	Boys	Girls	Boys
Intercept	3.43 [2.06, 4.80]	3.19 [2.10, 4.27]	2.17 [0.86, 3.49]	3.21 [2.19, 4.22]	3.10 [1.71, 4.49]	4.68 [3.68, 5.67]
Activity only vs. none	-0.05 [-0.13, 0.04]	-0.18 [-0.26, -0.10]^***^	0.16 [0.07, 0.24]^***^	0.15 [0.07, 0.22]^***^	0.15 [0.06, 0.23]^***^	0.19 [0.11, 0.26]^***^
Sleep only vs. none	-0.44 [-0.49, -0.40]^***^	-0.29 [-0.34, -0.24]^***^	0.49 [0.44, 0.53]^***^	0.33 [0.28, 0.38]^***^	0.54 [0.49, 0.58]^***^	0.37 [0.32, 0.42]^***^
Screen only vs. none	-0.23 [-0.38, -0.09]^**^	-0.12 [-0.33, 0.09]	0.10 [-0.04, 0.24]	0.04 [-0.16, 0.24]	0.15 [0.00, 0.29]	-0.08 [-0.28, 0.11]
Activity and sleep vs. none	-0.56 [-0.62, -0.49]^***^	-0.52 [-0.58, -0.46]^***^	0.65 [0.59, 0.71]^***^	0.56 [0.50, 0.61]^***^	0.75 [0.68, 0.82]^***^	0.57 [0.52, 0.63]^***^
Activity and screen vs. none	-0.29 [-0.48, -0.09]^**^	-0.59 [-0.82, -0.37]^***^	0.2 [0.02, 0.39]^*^	0.59 [0.38, 0.80]^***^	0.21 [0.01, 0.41]^*^	0.51 [0.30, 0.71]^***^
Sleep and screen vs. none	-0.81 [-0.87, -0.74]^***^	-0.51 [-0.60, -0.43]^***^	0.78 [0.71, 0.84]^***^	0.47 [0.40, 0.55]^***^	0.84 [0.78, 0.91]^***^	0.40 [0.33, 0.48]^***^
All three behaviours vs. none	-1.05 [-1.14, -0.96]^***^	-0.75 [-0.84, -0.66]^***^	0.99 [0.90, 1.07]^***^	0.75 [0.66, 0.83]^***^	1.03 [0.94, 1.13]^***^	0.66 [0.58, 0.75]^***^
Age	0.03 [-0.08, 0.14]	0.01 [-0.07, 0.10]	0.05 [-0.06, 0.16]	0.00 [-0.08, 0.08]	-0.03 [-0.14, 0.09]	-0.11 [-0.19, -0.03]^***^
Grade 7 vs. Grade 6	-0.06 [-0.19, 0.07]	-0.07 [-0.17, 0.04]	-0.07 [-0.19, 0.05]	0.02 [-0.08, 0.12]	-0.04 [-0.17, 0.08]	0.06 [-0.04, 0.16]
Grade 8 vs. Grade 6	0.11 [-0.12, 0.34]	-0.04 [-0.23, 0.14]	-0.3 [-0.52, -0.08]	-0.1 [-0.28, 0.07]	-0.27 [-0.5, -0.04]^*^	0.00 [-0.17, 0.17]
Neighbourhood SES index (2016)	-0.00 [-0.00, -0.00]^**^	-0.00 [-0.00, -0.00]^**^	0.00 [0.00, 0.00]^*^	0.00 [-0.00, 0.00]	0.00 [0.00, 0.01]^***^	0.00 [0.00, 0.00]^**^
Adjusted ICC	0.012	0.015	0.015	0.013	0.010	0.017

*Notes.* All models were adjusted for age, grade, and neighbourhood SES

^*^p < 0.05. ^**^p < 0.01. ^***^p < 0.001

We observed some sex differences in the associations between movement behaviours and well-being (Fig. 2a-c). For girls, associations with well-being were larger when they met recommendations for sleep, sleep and screen time, or for all movement behaviours. In contrast, for recommendations involving physical activity only, screen time only, physical activity and screen time, and physical activity and sleep, the associations with well-being outcomes were overall similar between boys and girls.

## Discussion

This study examined associations between adherence to Canada’s 24-Hour Guidelines and indicators of mental well-being among a large, population-based sample of adolescents in BC. Our findings provide insight into the extent of adherence to these guidelines and associations with positive and negative mental well-being outcomes, while also exploring sex differences. The majority of adolescents did not meet all movement recommendations. Only 4% adhered to all three guidelines, which aligns with findings from studies in other Canadian contexts [11, 46]. The adherence rate was highest for sleep (73%), followed by PA (26%). Adherence was lowest for screen time (14%), highlighting the ongoing challenge of reducing recreational screen time among adolescents, a trend that has been documented globally [19]. 

As expected, we found that adherence to multiple movement guidelines was associated with better mental well-being. Adolescents who met all three movement behaviour recommendations reported the highest levels of life satisfaction and optimism and the lowest levels of depressive symptoms. This finding is consistent with previous research demonstrating a dose-response relationship, where meeting more movement guidelines was associated with better physical and mental health outcomes [7, 11, 30]. Among adolescents who met only one recommendation, we found that adherence to the sleep guideline showed the strongest association with positive mental well-being, even when they did not meet the PA or screen time recommendations. This underscores the critical role of adequate sleep in adolescent mental well-being, supporting existing literature that emphasizes sleep as a key factor in emotional regulation and mental health and well-being [29, 30, 47]. We were not able to distinguish the strength of the associations between the combinations of movement behaviours among those who met two recommendations. Similarly, Janssen et al. also did not find a difference between combinations within those who met two recommendations [7]. 

Adherence rates differed for boys and girls in our study. Fewer girls (20%) met PA recommendations than boys (32%); fewer boys (11%) met screen time recommendations than girls (17%); and fewer girls (70%) met sleep recommendations than boys (76%). Such differences were also documented in previous literature [11, 16, 48, 49]. Sex differences in PA have been discussed through potential biological mechanisms (e.g., earlier physiological maturation in girls than boys) [50] and research suggests that girls are less likely to participate in organized sports than boys [51]. Research also suggests that girls tend to enjoy physical education in school less [52] and tend to receive less social support for PA than boys [53]. Sex differences in screen time have been less consistent and differed by activity in past studies. For example, excessive screen time was more common in girls when measures prioritized social activities (e.g., texting, hanging out with friends online) and social media [54], and more common in boys when they prioritized playing online/video games [55, 56]. Our study included social media usage as a measure, but not general socializing online (text messages or facetime), which may explain why girls had overall higher adherence levels than boys.

We found stronger associations between some movement behaviour patterns (sleep only, sleep and screen time, or all movement behaviours) and mental well-being among girls, indicating girls might benefit more from interventions targeting these behaviour combinations. Indeed, research suggests that social media use among adolescent girls is more strongly associated with negative mental health outcomes than it is for boys [57]. Halliday et al. (2019) found that adolescent girls reported less PA and worse mental health compared to boys and suggested that PA might partially explain sex differences in mental health [58]. Taken together, these sex differences highlight the importance of considering specific interventions and support when addressing movement behaviours and mental well-being among adolescent girls and boys.

Our findings have implications for public health policy and interventions. First, the low adherence to the screen time guideline underscores the need for targeted interventions to reduce recreational screen time among adolescents. A meta-analysis by Jones et al. found that interventions incorporating techniques like goal setting, goal review, and self-monitoring produced more significant effects on screen time reduction than interventions that did not incorporate these strategies [59]. A randomized controlled trial of the Active Teen Leaders Avoiding Screen-Time (ATLAS) intervention found significant screen-time reduction among adolescent boys (*N* = 351) [60]. Similarly, a cluster randomized controlled trial of the school-based obesity prevention program Nutrition and Enjoyable Activity for Teen Girls (NEAT Girls) with 357 adolescent girls found significant changes in screen time [61]. Regarding the potential of policy as a population-level intervention to regulate screen time, the parliament in Australia passed a bill in November 2024 - referred to as Online Safety Amendment (Social Media Minimum Age) 2024– that prevents adolescents under the age of 16 to access social media platforms [62]. Relatedly, in the context of BC, the Ministry of Education recently restricted the use of electronic devices and recreational screen time at school during school hours, limiting the use of devices to educational activities approved by an educator or school staff [63]. These policies are recent and their impact on adolescent screen time and potentially other movement behaviours has yet be examined in research.

Regarding interventions in the school setting, comprehensive school health promotion provides a suitable framework for promoting adherence to the 24-hour movement guidelines [64]. An umbrella review demonstrated that curriculum changes promoting PA, health education, and classroom breaks effectively reduced sedentary behaviours including recreational screen time (dos Santos et al., 2019). Schools are ideal contexts to integrate PA promotion through regular physical education and a built environment that supports adolescents to be physically active during recess and breaks [65]. There is limited evidence on interventions combining different aspects of movement behaviours [66]. However, some early evidence shows that multi-component interventions in high school can be useful to improve adherence to the 24-Hour Guidelines [65]. These programs can involve curricular (e.g., workshops and role modeling) and extracurricular (e.g., parental sessions) components [65]. 

### Limitations

The study has several strengths, including the use of population-based data. However, several limitations remain. Movement behaviours were self-reported by adolescents and therefore subject to social desirability and recall bias. Self-report data can furthermore introduce random measurement error and result in regression dilution bias [67]. PA was measured with one item and sleep was measured based on self-reported typical sleep and wake-up times. Future research needs to use multiple measures, including objective measures such as accelerometers to measure PA and sleep duration. Recreational screen time activities change constantly as new technologies emerge. It is possible that some types of recreational screen time were not captured in this study, due to activities which may not be captured in our screen time measure (e.g., activities on virtual reality devices). Furthermore, adolescents often engage in multiple screen-based activities simultaneously which can result in over-estimating their total screen time. The cross-sectional nature of the data precludes the ability to infer causality between movement behaviours and mental well-being. Reverse causality, whereby youth with higher levels of well-being may be more likely to be physically active, sleep well, and less likely to spend long hours in recreational screen time, cannot be ruled out. Future research needs to examine the association between meeting 24-hour guideless and mental well-being, using longitudinal designs. Finally, while sex differences were explored in our analysis, more explanatory analyses are needed to understand how sex and gender, as well as other social identities affect both movement behaviours and mental well-being among adolescents.

## Conclusion

In conclusion, this population-based study contributes to the growing body of literature on adherence to movement behaviour guidelines and the associations between adherence and mental well-being in adolescents. The findings highlight the importance of promoting adherence to all three components of the 24-Hour Guidelines, particularly in reducing screen time and ensuring adequate sleep, to support the mental well-being of adolescents. Tailored interventions that consider sex differences may be especially beneficial in addressing the unique needs of boys and girls in promoting their overall well-being.

## Electronic supplementary material

Below is the link to the electronic supplementary material.

Supplementary Material 1

## Data Availability

Data included in this study are stored with Population Data BC at the University of British Columbia https://www.popdata.bc.ca/researchers, and can only be accessed upon approval from data stewards. Self-report questions used in this study are available from the main author upon request.
