# Supplementary material for: Adherence to 24-hour movement guidelines and associations with mental well-being: a population-based study with adolescents in Canada
Source: BMC Public Health. 2025 Mar 6;25:749. doi: 10.1186/s12889-025-21857-7 (PMC11884116; doi:10.1186/s12889-025-21857-7)
Supplement: Supplementary file 1 — Supplementary Material 1 [file 12889_2025_21857_MOESM1_ESM.docx]

**Appendix. Missing data analysis**

Comparison of sample characteristics between participants without and with missing data is shown in Table A1. In the imputation model, we included the exposure (categorical), outcome (depressive symptoms, continuous), all covariates in the primary models, and the following auxiliary variables: first language (English vs non-English), school ID, city, optimism score, satisfaction with life score, empathy score, anxiety score, prosocial behaviour score, measures for connectedness, peer belonging score, school support, school belonging, bullying, responsible decision-making, self-awareness, self-regulation, perseverance, assertiveness, neighbourhood ID, as individual movement behaviours. 10 imputed dataset were generated using multiple imputation by chain equation (MICE) with predictive mean matching for continuous variables, logistic regression for binary variables, and polynomial regression for categorical variables.^1^ We ran linear mixed models in each computed data and pooled the results using Rubin’s rule for each sex separately.^2^

*Table A1. Sample characteristics between participants without and with missing data*

|  |  | Total (N=26974) |  | Girls (N=13053) |  | Boys (N=13921) |  |
| --- | --- | --- | --- | --- | --- | --- | --- |
|  |  | Complete cases | Missing | Complete cases | Missing | Complete cases | Missing |
| N (%) |  | 24231 (89.8) | 2743 (10.2) | 11802 (90.4) | 1251 (9.6) | 12429 (89.3) | 1492 (10.7) |
| Grade (%) | Grade 6 | 6280 (25.9) | 834 (30.4) | 3143 (26.6) | 395 (31.6) | 3137 (25.2) | 439 (29.4) |
|  | Grade 7 | 4673 (19.3) | 487 (17.8) | 2258 (19.1) | 235 (18.8) | 2415 (19.4) | 252 (16.9) |
|  | Grade 8 | 13278 (54.8) | 1422 (51.8) | 6401 (54.2) | 621 (49.6) | 6877 (55.3) | 801 (53.7) |
| Age, mean (SD) | | 13.31 (0.87) | 13.25 (0.91) | 13.29 (0.87) | 13.20 (0.91) | 13.33 (0.87) | 13.29 (0.90) |
| Optimism, mean (SD) | | 3.46 (0.95) | 3.44 (0.96) | 3.31 (0.97) | 3.33 (0.96) | 3.60 (0.92) | 3.53 (0.95) |
| SWL, mean (SD) | | 3.64 (0.97) | 3.61 (0.96) | 3.46 (1.02) | 3.46 (0.99) | 3.81 (0.90) | 3.73 (0.91) |
| Depression, mean (SD) | | 3.02 (1.01) | 3.03 (0.99) | 3.25 (1.00) | 3.28 (0.99) | 2.80 (0.97) | 2.84 (0.95) |
| SES index 2016, mean (SD) | | 110.70 (13.22) | 110.00 (13.19) | 110.74 (13.16) | 110.58 (13.37) | 110.66 (13.27) | 109.51 (13.02) |

*Table A2. Comparison between main results and multiple imputation analyses (depressive symptoms)*

|  | **Main analyses** | | **Multiple imputation (m=10)** | |
| --- | --- | --- | --- | --- |
| Est. | **Girls** | **Boys** | **Girls** | **Boys** |
| **Intercept** | 3.43 [2.06, 4.80] | 3.19 [2.10, 4.27] | 3.56 [2.22 ,4.90] | 2.84 [1.73 ,3.94] |
| **Activity only vs none** | -0.05 [-0.13, 0.04] | -0.18 [-0.26, -0.10]^***^ | -0.06 [-0.17 ,0.05] | -0.15 [-0.24 ,-0.05]^**^ |
| **Sleep only vs none** | -0.44 [-0.49, -0.40]^***^ | -0.29 [-0.34, -0.24]^***^ | -0.41 [-0.46 ,-0.36]^***^ | -0.26 [-0.32 ,-0.20]^***^ |
| **Screen only vs none** | -0.23 [-0.38, -0.09]^**^ | -0.12 [-0.33, 0.09] | -0.26 [-0.42 ,-0.10]^**^ | -0.13 [-0.36 ,0.10] |
| **Activity and sleep vs none** | -0.56 [-0.62, -0.49]^***^ | -0.52 [-0.58, -0.46]^***^ | -0.51 [-0.58 ,-0.44]^***^ | -0.48 [-0.55 ,-0.42]^***^ |
| **Activity and screen vs none** | -0.29 [-0.48, -0.09]^**^ | -0.59 [-0.82, -0.37]^***^ | -0.24 [-0.45 ,-0.04]^*^ | -0.45 [-0.73 ,-0.17]^***^ |
| **Sleep and screen vs none** | -0.81 [-0.87, -0.74]^***^ | -0.51 [-0.60, -0.43]^***^ | -0.74 [-0.82 ,-0.67]^***^ | -0.44 [-0.55 ,-0.32]^***^ |
| **All three behaviours vs none** | -1.05 [-1.14, -0.96]^***^ | -0.75 [-0.84, -0.66]^***^ | -0.98 [-1.08 ,-0.88]^***^ | -0.66 [-0.78 ,-0.54]^***^ |
| **Age** | 0.03 [-0.08, 0.14] | 0.01 [-0.07, 0.10] | 0.02 [-0.09 ,0.13] | 0.05 [-0.05 ,0.14] |
| **Grade 7 vs Grade 6** | -0.06 [-0.19, 0.07] | -0.07 [-0.17, 0.04] | -0.02 [-0.15 ,0.10] | -0.10 [-0.22 ,0.01] |
| **Grade 8 vs Grade 6** | 0.11 [-0.12, 0.34] | -0.04 [-0.23, 0.14] | 0.14 [-0.09 ,0.36] | -0.12 [-0.31 ,0.07] |
| **Neighbourhood SES index (2016)** | -0.00 [-0.00, -0.00]^**^ | -0.00 [-0.00, -0.00]^**^ | -0.00 [-0.00, -0.00]^*^ | -0.00 [-0.00, -0.00]^**^ |

*Notes.* All models were adjusted for age, grade, and neighbourhood SES index (2016). We present the estimates for these variables in the table as well.

^*^p<0.05. ^**^p<0.01. ^***^p<0.001

**Reference**

1. Buuren S van, Groothuis-Oudshoorn K. mice: Multivariate Imputation by Chained Equations in R. Journal of Statistical Software. 2011;45(3):1–67.

2. Rubin DB. Multiple Imputation for Nonresponse in Surveys [Internet]. 1st ed. Wiley; 1987 [cited 2024 Nov 11]. (Wiley Series in Probability and Statistics). Available from: https://onlinelibrary.wiley.com/doi/book/10.1002/9780470316696
